# Change in Body Mass Index After Breast Reconstruction and Associated Complications

**Published:** 2015-09-28

**Authors:** Matthew A. Applebaum, Bradford T. Miller, Jonathan Lopez, Erin L. Doren, Christine Laronga, Paul D. Smith

**Affiliations:** ^a^University of South Florida Morsani College of Medicine, Tampa; ^b^Division of Plastic Surgery, Department of Surgery, University of South Florida, Tampa; ^c^Comprehensive Breast Program, H. Lee Moffitt Cancer Center, Tampa, Fla

**Keywords:** breast reconstruction, obesity, breast cancer, breast cancer treatment, breast surgery complication

## Abstract

**Objective:** The incidence and prevalence of breast cancer continue to rise. Therapies may contribute to patient weight gain. Obesity, a major predictor of surgical complications, may affect reconstructive outcome. The goal of this study was to quantify weight gain/change after the diagnosis and treatment of breast cancer in women choosing reconstruction after mastectomy. **Methods:** Retrospective review of patients undergoing mastectomy with reconstruction at a dedicated Cancer Center from 1996 to 2011 was conducted. Patient demographics, body mass index (BMI), and surgical complications were reported. Patients were stratified as normal weight (BMI <25 kg/m^2^) and overweight/obese (BMI >25 kg/m^2^). Body mass index at the time of mastectomy was compared with BMI postreconstruction. **Results:** A total of 443 patients had mastectomy and reconstruction. Forty-nine percent of patients were classified as normal weight (BMI <25 kg/m^2^) at the time of mastectomy and 51% as overweight/obese (body mass index > 25 kg/m^2^). Mean body mass index at the time of mastectomy was 26.1 kg/m^2^ (4.9 SD) and 26.4 kg/m^2^ (5.1 SD) at the final follow-up. Median follow-up time was 2.7 years (range <1 to 15 years). There was no statistically significant change in BMI before and after cancer treatment (*P* > .05). However, overweight/obese patients with complications were more likely to require an unanticipated return to the operating room (*P* = .0124). **Conclusions:** Despite the stress of breast cancer diagnosis, surgical treatment, and reconstruction, we find that patients' weight does not change significantly over time. Overweight and obese patients are not always at higher risk for surgical complications but may have more severe complications when they do occur.

Breast cancer is the most frequently diagnosed cancer in women.^1^ It is estimated that in 2015 there will be approximately 231,840 new cases of invasive breast cancer diagnosed in the United States.^1^ Now more than one third of US adults are classified as obese.^2^ In 2014, the total number of performed breast reconstructive procedures increased by 7%.^3^ As the number of breast cancer–related reconstructions increases, so does emphasis on positive reconstructive outcome. Selecting the best reconstructive option includes consideration of the patient's pretreatment body habitus.

Previous studies have indicated that patients may be more likely to experience an increase in body mass index (BMI) after receiving the diagnosis and treatment of breast cancer.^4^^-^6 Leading treatments, such as chemotherapy, have been strongly associated with increased patient weight.^7^^-^11 Obesity has been associated with an increased recurrence and mortality from breast cancer.^12^^-^15 In addition, obesity is a known predictor of complications in breast surgery and reconstruction.^1^^,^^16^^,^^17^ If patients’ weight increases significantly as the result of breast cancer diagnosis or treatment, surgical complications may occur and reconstructive outcomes negatively affected.

This study investigates changes in patients’ BMI after the diagnosis and treatment of breast cancer. In addition, we assess outcomes and complications associated with breast cancer reconstruction in the normal and overweight/obese BMI categories at a large-volume cancer center.

## METHODS

This is a retrospective review of a prospectively gathered breast reconstruction database. After internal review board approval, the database was searched for patients having undergone mastectomy with reconstruction (immediate or delayed) between June 1996 and August 2011. Patient demographics were collected and included age, stage at diagnosis, adjuvant treatment, surgical treatment, and complications. Patients’ BMI at the time of mastectomy was compared with BMI at the date of their last follow-up, after mastectomy, and breast reconstruction (range, <1 to 15 years). Percent change in BMI was calculated from this data set.

Outcomes and complications of breast reconstruction were recorded and compared between 2 BMI categories, normal weight (BMI <25 kg/m^2^) and overweight/obese (BMI >25 kg/m^2^). Variables related to breast reconstruction outcomes included infection, skin necrosis, seroma, hematoma, contour deformity, asymmetry, wound dehiscence, hypertrophic scarring, malposition of prosthesis, implant rupture, autologous flap revision, flap loss, hernia, capsular contracture, nipple ischemia, and any complication requiring a return to the operating room (OR). Complications were compared between the BMI categories.

The statistical analysis for the normally distributed BMI change was performed using a 2-sided 1-sample *t* test. *P* values were calculated using the χ^2^ test utilizing the exact method with Monte Carlo estimation. A *P* value of .05 indicated statistical significance.

## RESULTS

A total of 443 patients were identified as having undergone mastectomy with breast reconstruction between 1996 and 2011. For 440 patients, BMI data were available (99%). The mean age at the time of presentation was 50.2 years (range, 18–82 years). A total of 780 mastectomies with reconstruction (106 unilateral, 337 bilateral) were performed. The most common reconstruction types included tissue expander with implant reconstruction (62.8% of breasts), latissimus flap with prosthesis (14.0%), and abdominally based flaps (13.6%).

A total of 218 patients (49%) were classified as normal weight (BMI <25 kg/m^2^) at the time of mastectomy, with 225 (51%) patients classifying as overweight/obese (BMI >25 kg/m^2^). Mean BMI at the time of presentation was 26.1 kg/m^2^ (4.9 SD) and 26.4 kg/m^2^ (5.1 SD) at final follow-up (Table 1). The median follow-up time was 2.7 years (range, 0.02–14.98 years).

**Figure 1:**
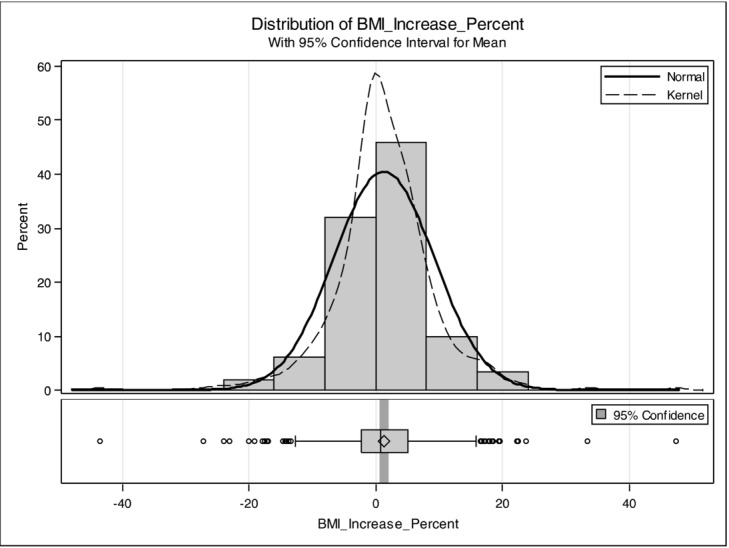
The percentage change in BMI was compared with the percentage of patient distribution. Changes in BMI are plotted as a percentage change. The mean change in BMI was 1.4% with a normal distribution and a range of −43.5% to 47.4%. BMI indicates body mass index.

**Figure 2:**
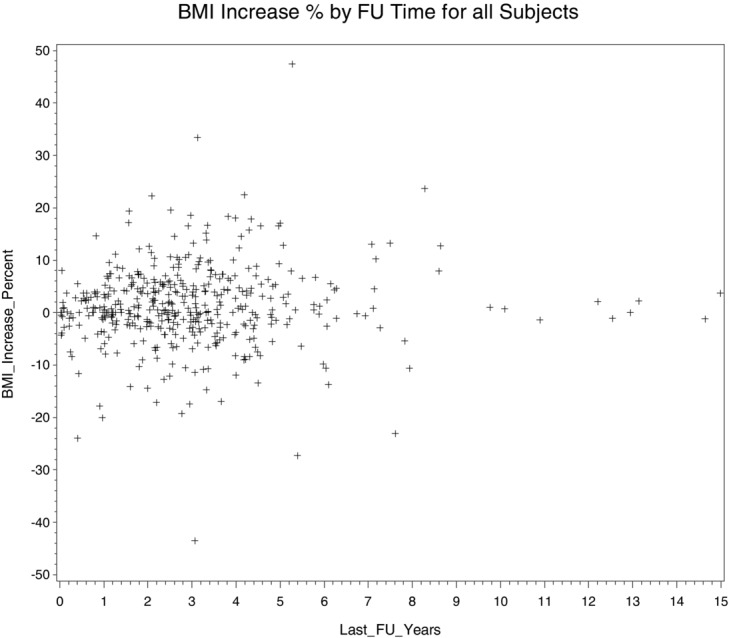
Percentage increase in BMI versus the length of the last follow-up. BMI indicates body mass index.

Patients were stratified on the basis of length of follow-up, with 57 patients followed up for less than 1 year, 88 patients 1 to 2 years, 103 patients 2 to 3 years, 89 patients 3 to 4 years, and 103 patients more than 4 years. For all groups, there were no statistically significant changes in BMI (*P* > .05). Changes in BMI are plotted as a percentage change. The mean change in BMI was 1.4% with a normal distribution and a range of −43.5% to 47.4% (Fig. 1). Percent change in BMI has been plotted by the time of the last follow-up (Fig. 2). BMI even on long-term follow-up does not change significantly.

A total of 245 patients (55%) experienced at least 1 major or minor complication. The most common complications were minor: fat necrosis (18%), infection (13%), epidermolysis (9.3%), and mastectomy skin necrosis (8.8%). The overweight/obese group had a significantly higher prevalence of diabetes (6.3% vs 0.9%; *P* = .0036) and hypertension (26.7% vs 11.1%; *P* < .0001). The overweight/obese group was also more likely to receive neoadjuvant chemotherapy (16.2% vs 4.8%; *P* = .0005), possibly due to presentation at a later stage, but no significant differences were identified. The overweight/obese group was found to be significantly older than the normal weight group (*P* = .0005). The overweight/obese group had a mean age of 50.65 years and a range of 30 to 80 years compared with the normal weight group's mean age of 48.33 years and a range of 18 to 75 years. Other comorbid conditions, including smoking, history of breast cancer, adjuvant chemotherapy, and history of postsurgical radiation, were found to be similar between the BMI groups.

There were no significant differences identified in the incidence of any individual complication between the BMI groups. The incidence of complications was similar between the BMI categories (overweight/obese, 56.9%; normal weight, 53.7%; *P* = .4918). When assessing complications per breast, instead of per patient, overweight/obese patients with a complication were significantly more likely to require an unanticipated return to the OR (overweight/obese, 58.1%; normal weight, 43.5%; *P* = .0124).

## DISCUSSION

Reports from the literature, as well as subjective complaints from our patients, led us to hypothesize that women experience an increase in weight after breast cancer diagnosis and surgical treatment. This is theorized to be secondary to factors such as stress, endocrine therapy, chemotherapy, and surgery. This retrospective review of patient changes in BMI, before and after the surgical treatment of breast cancer, reveals that patients’ BMI actually remains stable. Contrary to our hypothesis, there was no significant gain in weight in this specific population (women having undergone mastectomy with reconstruction), only a 1.4% mean increase in BMI, a clinically insignificant change.

Previous literature has shown that after the diagnosis and treatment of breast cancer, with chemotherapy, patients often have an increased BMI.^9^ However, a significant weight gain directly related to treatment is not shown. Furthermore, prospective studies show that the likelihood of weight gain was increased after chemotherapy treatment in patients who lead sedentary lifestyles.^4^ Sedentary time was noted to be elevated throughout the first year following treatment and was seen to be prevalent in patients who were overweight prior to treatment.^4^ Our study attempted to see if there was a significant increase in weight and BMI after treatment. We found that, compared with previous literature, in the absence of cofactors to weight gain, patients appeared to remain at the same relative weight and BMI.

Postoperative complications have been noted to increase in the overweight and obese populations. Obesity has been shown to increase complication rates by 18.3% in patients who elect to have breast surgery.^1^ A cohort study showed patients undergoing elective breast surgery (breast reduction, reconstruction, mastopexy with augmentation, mastopexy alone, and augmentation alone) were at higher risk for complications if they were obese compared with control groups.^1^ Our study showed that when comparing normal weight patients with overweight/obese patients, there were no significant differences in complication rate. However, when looking at complications by breast, overweight/obese patients with a complication were more likely to require a return to the OR, indicating a major complication requiring surgical management versus a minor complication.

This study does present some limitations. First, BMI was compared at only 2 time points, at mastectomy and last follow-up visit. Had BMI been plotted at each year's visit, perhaps we may find a different trend, such as weight gain immediately following reconstruction subsequently trending back to presurgical weight. We did find that when follow-up was short, 1 to 2 years after mastectomy and reconstruction, BMI did not on average change, leading us to believe that in fact there would not be any significant BMI change even if plotted per patient over time. The other limiting factors of this study are that complications associated with BMI category did not account for confounding variables and that we did not have a control group of patients who did not have reconstruction. Had a regression analysis been performed we might have found more significance in weight-related complications. Patients choosing reconstruction may be different with regard to body image and comorbid conditions from those electing not to have reconstruction. Patients not having reconstruction may have a higher BMI and live a more sedentary lifestyle at baseline that may lead to an increased propensity for weight gain with breast cancer treatment. This study, however, offers insight into a large reconstructive practice at a dedicated cancer center. A multi-institution study may provide more generalizable data.

## CONCLUSION

Despite the stress of breast cancer diagnosis and treatment, we find that patients’ weight does not change significantly after the surgical treatment of breast cancer and reconstruction. We therefore choose to base our reconstructive plan on preoperative weight, knowing that a patient will not likely gain or lose weight over time. We additionally find that overweight and obese patients are not always at higher risk for surgical complications but may have a more severe complication when they do occur.

## Figures and Tables

**Table 1:** Statistical analysis for the normally distributed weight change performed using a 2-sided 1-sample t test*

Variable	*n*	Minimum	Median	Maximum	Mean	SD
BMI	439	18.6	25.1	48.7	26.0	4.8
Last_BMI	16.6	25.7	45.2	26.3	4.8	
BMI_Increase_Percent	438	−43.5	0.8	47.4	1.3	7.9

*Mean and median change in weight were less than 1.5% and insignificant (*P* < .05) for the group and when stratified by years of follow-up. Contrary to our hypothesis, the mean and the median change in weight were insignificant. BMI indicates body mass index.
